# Hormonal Contraceptive Use, Stress Disorders, and Cardiovascular and Thrombotic Risk in Women

**DOI:** 10.1001/jamanetworkopen.2025.51878

**Published:** 2026-01-02

**Authors:** Jordan L. Thomas, Robyn A. Ellis, Krystel AbiKaram, Shady Abohashem, Anahita Dua, Emily S. Lau, Maureen J. MacDonald, Karen K. Miller, Suzanne L. Pineles, Rachel P. Rosovsky, Ahmed Tawakol, Michael T. Osborne, Antonia V. Seligowski

**Affiliations:** 1 Behavioral Science Division, National Center for PTSD, VA Boston Healthcare System, Boston, Massachusetts; 2 Department of Psychiatry, Boston University Chobanian & Avedisian School of Medicine, Boston, Massachusetts; 3 Department of Psychology, University of Kansas, Lawrence; 4 McLean Hospital, Belmont, Massachusetts; 5 Department of Psychiatry, Harvard Medical School, Boston, Massachusetts; 6 Cardiovascular Imaging Research Center, Massachusetts General Hospital, Boston; 7 Vascular Center, Massachusetts General Hospital, Boston; 8 Cardiology Division, Department of Medicine, Massachusetts General Hospital, Boston; 9 Department of Kinesiology, McMaster University, Hamilton, Ontario, Canada; 10 Neuroendocrine Unit, Massachusetts General Hospital and Harvard Medical School, Boston; 11 Women’s Health Sciences Division, National Center for PTSD, VA Boston Healthcare System, Boston, Massachusetts; 12 Division of Hematology, Department of Medicine, Massachusetts General Hospital and Harvard Medical School, Boston

## Abstract

**Importance:**

Stress and its psychiatric consequences—including depression, anxiety, and posttraumatic stress disorder (PTSD)—are pertinent to women’s cardiovascular health, but research on intersections with relevant sex-specific factors (eg, hormonal contraceptives) is lacking.

**Objective:**

To examine whether stress-related psychiatric diagnoses moderate associations between hormonal contraceptive use and cardiovascular and thrombotic risk.

**Design, Setting, and Participants:**

This retrospective cohort study included electronic health record data collected from a US hospital-based biobank and analyzed from May 2, 2024, to November 3, 2025. Participants were women aged 18 to 55 years who consented into the biobank before or on September 12, 2020.

**Exposures:**

Lifetime history of stress-related psychiatric disorders, including depression (major depressive disorder), anxiety (generalized anxiety disorder, social anxiety disorder, or panic disorder), and PTSD, defined by *International Statistical Classification of Diseases and Related Health Problems, Tenth Revision* (*ICD-10*) codes and analyzed as separate diagnoses, and lifetime history of combined hormonal contraceptive use, defined by RxNorm codes.

**Main Outcomes and Measures:**

The primary outcomes were major adverse cardiovascular events (MACE; defined as *ICD-10* codes for infarction, unstable angina, heart failure, coronary revascularization, peripheral vascular disease, peripheral revascularization, stroke, and/or transient ischemic attack) and deep-vein thrombosis (DVT). Three 2-step hierarchical logistic regressions per outcome were conducted.

**Results:**

In this sample of 31 824 women (mean [SD] age, 38.5 [10.6] years), over one-third (11 950 women [37.6%]) had hormonal contraceptive use history, and stress-related disorders were common (depression, 9116 women [28.5%]; anxiety, 3533 women [11.1%]; PTSD, 1992 women [6.3%]). Associations were mixed across the stress-related disorders, in that depression and anxiety did not moderate associations between contraceptive use and MACE or DVT. In contrast, PTSD modified the association between contraceptive use and MACE but not that between contraceptive use and DVT. Analyses stratified by PTSD status found that only women without PTSD using contraceptives had lower odds for MACE (odds ratio, 0.69; 95% CI, 0.87-3.24). The odds ratio for MACE among women with PTSD was greater than 1, but the finding was not statistically significant (odds ratio, 1.68; 95% CI, 0.87-3.24).

**Conclusions and Relevance:**

In this retrospective cohort study, combined hormonal contraceptive use was associated with lower cardiovascular risk in women regardless of depression or anxiety. These protective associations did not extend to women with PTSD, suggesting that there are unique cardiovascular processes in the context of this stress-related disorder and hormonal contraceptive use that warrant further research.

## Key Points

**Question:**

Do associations between combined hormonal contraceptive use and cardiovascular and thrombotic risk differ if women have stress-related psychiatric conditions (depression, anxiety, or posttraumatic stress disorder [PTSD])?

**Findings:**

In this cohort study of 31 824 women, hormonal contraceptive use was associated with lower odds of major adverse cardiovascular events in women irrespective of depression or anxiety history. Contraceptive use was associated with lower cardiovascular risk among women without PTSD, but not women with PTSD.

**Meaning:**

These findings suggest that contraceptive use is associated with cardiovascular health outcomes in women with depression and anxiety, but not PTSD.

## Teaser

This cohort study assesses whether stress-related psychiatric diagnoses moderate the associations between hormonal contraceptive use and cardiovascular risk.

## Introduction

Over 400 000 women in the US die annually from cardiovascular disease (CVD), the nation’s leading cause of mortality.^1^ Stress is increasingly recognized as a CVD risk factor,^2,3^ and stress-related psychiatric disorders such as depression, anxiety, and posttraumatic stress disorder (PTSD), all of which are conditions common after stressful and traumatic life events, are overrepresented in women, highly comorbid with one another,^4^ and collectively implicated in cardiovascular health.^5^ Furthermore, women exhibit distinct cardiovascular risk profiles and clinical manifestations^6,7,8,9,10,11^ with certain conditions, such as deep vein thrombosis (DVT), more common in premenopausal women compared with age-matched men.^12,13,14^ These disparities have spurred experts to call for greater attention to sex-specific factors that may contribute to adverse cardiovascular health in women.^15^

An understudied sex-specific factor is hormonal contraception, which is used by approximately 9.1 million women in the US.^16^ Hormonal contraceptives introduce exogenous hormones of varying compositions and doses and suppress endogenous estradiol and progesterone levels. While endogenous sex hormones in naturally cycling women (ie, those not using hormonal contraception) appear to be protective against CVD, the CVD-relevant effects of hormonal contraceptive use are mixed.^17^ For example, while some studies found elevated cardiovascular (eg, stroke or myocardial infarction) and thrombotic risk in women using oral hormonal contraception,^18,19,20^ others localized this to women with additional risk factors (eg, those who smoke) or found no association.^21,22,23,24^

Mixed findings may be due, in part, to variability in hormonal components that make up different contraceptives. Four generations of hormonal contraceptives exist, distinguished largely by their ratios of ethinyl estradiol to progestin.^25^ First-generation and second-generation contraceptives are characterized by higher estrogen doses than third-generation and fourth-generation contraceptives, which typically include a smaller amount of estrogen combined with newer progestins. Research suggests that these generations differentially impact vascular function and thrombotic risk, with earlier variants more commonly associated with impaired endothelial function and thrombosis risk than later versions.^26,27,28^

A substantial limitation to this research is that it has almost exclusively been conducted in young, healthy adults, with little known about these associations in the context of psychiatric disorders that disproportionately impact women (eg, anxiety and depression). To our knowledge, no research has tested the combined associations of hormonal contraceptives and stress-related disorders with cardiovascular or thrombosis risk. Work in nonpsychiatric populations has found that different formulations of hormonal contraceptives have negative associations with autonomic and vascular function and thrombosis risk^25^—pathophysiological processes implicated in stress-related disorders.^29^ A natural extension is to examine whether similar associations exist in women with stress-related disorders.

As such, we leveraged a hospital-based biobank to investigate combined hormonal contraceptive use and cardiovascular and thrombotic risk in women with and without stress-related disorders. We hypothesized that compared with women without these psychiatric conditions, women with a lifetime history of depression, anxiety, or PTSD who were hormonal contraceptive users would bear greater risk of major adverse cardiovascular events (MACE) and DVT. Exploratory analyses considered contraception according to generation. We hypothesized that women with stress-related disorders using earlier generation forms of contraception (eg, second-generation) would demonstrate elevated MACE risk, while women with stress-related disorders using third-generation contraception would demonstrate elevated DVT risk.^25^

## Methods

### Participants and Materials

This cohort study used data from the Mass General Brigham Biobank, a biorepository of patients recruited throughout the Mass General Brigham health care system (eg, Massachusetts General Hospital, McLean Hospital). Patients may opt to sign a data and sample use agreement and consent to researchers utilizing their deidentified health records, which include data (eg, diagnoses) from the electronic health record (EHR), blood samples, and surveys. Patients can consent into the biobank at any point in their clinical care, at which point their entire EHR is accessible to researchers, including information entered prior to the date of consent. Consented patients can withdraw their biobank consent at any time. Procedures were approved by the Mass General Brigham institutional review board, and we have structured our reporting to be consistent with guidelines set forth by the Strengthening the Reporting of Observational Studies in Epidemiology (STROBE) reporting guideline for cohort studies.^30^

For this project, we restricted the sample to women aged 18 to 55 years, as older women would be more likely to have ceased contraceptive use due to menopause. The overall analytic sample included women in this age range who consented into the biobank on or before September 12, 2020, and had active consent through March 13, 2025; see the eFigure in Supplement 1 for details on the study cohort.

Clinical and demographic data—including participants’ self-identified race and ethnicity—were obtained from the EHR. Data on race and ethnicity were included in this analysis because of their potential associations with stress disorders and both MACE and DVT. *International Statistical Classification of Diseases and Related Health Problems, Tenth Revision* (*ICD-10*) codes were used to determine diagnoses of stress-related disorders, MACE, and DVT. Hormonal contraceptive use was determined by RxNorm code, which captures prescribed medications. Individuals needed to have at least 1 *ICD-10* and/or RxNorm code in their EHR to be considered a positive case, as done in previous biobank studies of psychiatric diagnoses^31^; individuals without relevant codes were coded as negative for that study variable. Stress-related disorders included depression (major depressive disorder, single episode and recurrent), anxiety (generalized anxiety disorder, social anxiety disorder, or panic disorder), and PTSD.

To ensure that contraceptive use and stress-related diagnoses preceded MACE and DVT, we used date of participant consent into the biobank to address temporality. We required the first date of the relevant *ICD-10* and/or RxNorm code to be present in the EHR prior to date of consent for the stress-related disorders and contraceptive use and after consent date for outcomes (MACE or DVT). As seen in the eFigure in Supplement 1, women with *ICD-10* and/or RxNorm dates that did not meet these criteria (eg, MACE prior to consent, initiated contraceptive use after consent) were removed from analyses to control for potential reverse causality.

Consistent with prior work,^32,33^ MACE was coded as the presence of any of the following: myocardial infarction, unstable angina, heart failure, coronary revascularization, peripheral vascular disease, peripheral revascularization, stroke, and transient ischemic attack. DVT was coded as the presence of an acute embolism and thrombosis of deep veins of a lower extremity. Women with RxNorm codes indicating combination hormonal birth control (eg, ethinyl estradiol and norgestimate or mestranol and norethindrone) were classified as contraceptive users; women with progestin-only contraceptives (eg, levonorgestrel) and histories of only noncombination methods were excluded. For exploratory analyses, users were also categorized by contraception generation (eTable in Supplement 1).

We also included information from the EHR on potential confounders (covariates) relevant to each outcome (eg, hypertension for MACE or cancer for DVT). Medical conditions were coded as present if an individual had a record of a diagnosis in their EHR, and absent if they did not. The only exceptions to this were smoking history and obesity, both of which were derived from an optional survey completed during biobank enrollment. Individuals reported their height and weight (which was used to calculate body mass index [calculated as weight in kilograms divided by height in meters squared], later dichotomized into obese vs not) and if they had or had not smoked at least 100 cigarettes in their lifetime. For those without survey data, information on past or current tobacco smoking and obesity status was collated from health history data from the EHR, such that individuals with records indicative of tobacco smoking and obesity were coded as positive on those variables. For individuals without survey or EHR data on smoking and obesity (12%-14% missingness across variables), multiple imputation was used.

### Statistical Analysis

For our primary analyses, we conducted three 2-step hierarchical logistic regressions per outcome. In the first step, we included main effects of preconsent hormonal contraceptive use and 1 of the 3 discrete stress-related disorders separately on postconsent MACE and DVT, as well as a moderation effect of each stress-related disorder on associations between contraceptive use and the cardiovascular and thrombotic events. As our primary interest was in the moderation association, and consistent with testing these effects in behavioral health research,^34,35^ we do not interpret main associations here. In the second step, we added disease-related confounds of relevance to each clinical outcome (covariates), including smoking, hypertension, hyperlipidemia, and diabetes for MACE and cancer, obesity, thrombophilia, and antiphospholipid syndrome for DVT. For significant moderation association across the steps (where step 1 is unadjusted and step 2 includes adjustment for clinical covariates), we stratified by psychiatric disorder status and examined associations between contraception and MACE and DVT among women with (vs without) each stress-related disorder. To address potential bias from missing data, we utilized multiple imputation. Missing values on covariates were imputed 14 times (reflecting the percentage of individuals with any missing data^36^) using logistic regression via the mice package in R statistical software version 4.4.1 (R Project for Statistical Computing); pooled model estimates are reported in the text and tables.

We adjusted for multiple comparisons using the Bonferroni correction per family of analyses, where outcome functioned as a family. As we conducted 3 separate hierarchical regressions per family, moderation associations were considered statistically significant at *P* < .017 (α = .05/3).

Next, exploratory analyses disaggregated the hormonal contraceptive into discrete generations to explore whether specific forms contributed to additional risk. Because 3084 women had histories of using multiple hormonal contraceptives spanning generations, these analyses were conducted only among 8109 women reporting a single generation type prior to consent. Second-generation was the reference group for MACE, whereas third-generation was the reference group for DVT (given higher MACE and DVT risk in these groups, respectively).^25^ As with the primary analyses, three 2-step hierarchical logistic regressions per outcome were conducted. Due to the exploratory nature of these analyses, we did not adjust for multiple comparisons.

## Results

The study included 31 824 women (mean [SD] age, 38.5 [10.6] years) (Table 1). Stress-related disorders were common; depression was the most prevalent (9116 women [28.5%]), followed by anxiety (3533 women [11.1%]) and PTSD (1992 women [6.3%]). In the cohort, 467 women (1.5%) had MACE and 297 (0.9%) had DVT. Over one-third of the sample (11 950 women [37.6%]) had codes indicating combined hormonal contraceptive use prior to consent. When contraceptive generations were classified according to whether women had ever used a particular type, third-generation (5607 women [17.6%]) and second-generation (3834 women [12.0%]) methods were the most well-represented. A similar pattern held when examining the subsample of women with a single generation history.

**Table 1.** Participant Characteristics

Characteristics	Participants, No. (%)		
	Overall (N = 31 824)	Contraceptive use (n = 11 950)	No contraceptive use (n = 19 874)
Age, mean (SD), y^a^	38.5 (10.6)	35.7 (9.4)	40.3 (10.9)
Race			
American Indian or Alaska Native	59 (0.2)	16 (0.1)	43 (0.2)
Asian	1381 (4.3)	408 (3.4)	973 (4.9)
Black or African American	1911 (6.0)	758 (6.3)	1153 (5.8)
Multiracial	457 (1.4)	197 (1.6)	260 (1.3)
Native Hawaiian or Pacific Islander	15 (>0.1)	5 (>0.1)	10 (0.1)
White	25 054 (78.7)	9395 (78.6)	15 659 (78.8)
Other^b^	1891 (5.9)	910 (7.6)	981 (4.9)
Ethnicity			
Hispanic	1297 (4.1)	694 (5.8)	603 (3.0)
Non-Hispanic	27 532 (86.5)	10 240 (85.7)	17 292 (87.0)
Depression	9116 (28.5)	4077 (34.1)	5039 (25.4)
Anxiety	3533 (11.1)	1876 (15.7)	1657 (8.3)
Posttraumatic stress disorder	1992 (6.3)	856 (7.2)	1136 (5.7)
Major adverse cardiovascular events	476 (1.5)	149 (1.2)	327 (1.6)
Deep vein thrombosis	297 (0.9)	88 (0.7)	209 (1.1)
Combined hormonal contraceptive use^c^	11 950 (37.6)		
First-generation	4127 (13.0)	4127 (34.5)	NA
Second-generation	3834 (12.0)	3834 (32.1)	NA
Third-generation	5607 (17.6)	5607 (46.9)	NA
Fourth-generation	1417 (4.5)	1417 (11.9)	NA

Abbreviation: NA, not applicable.

^a^ Age range, 18 to 55 years

^b^ Other race included any race not otherwise specified.

^c^ Contraceptive generations as depicted here are not mutually exclusive and represent ever using a particular generation.

### Associations Between Stress-Related Disorders, Contraceptive Use, and MACE and DVT

Tables 2, 3, and 4 detail results of the primary analyses examining associations between contraceptive use and cardiovascular and thrombotic outcomes as moderated by each stress-related disorder. While estimates for unadjusted models are available in the tables, we focus our interpretation here on covariate-adjusted models. Significant moderation associations were probed by stratified analyses (Table 5).

**Table 2.** Associations of Depression and Contraceptive Use With MACE and DVT

Outcome, step, and variable	OR (95% CI)	*b*	*P* value
MACE (n = 27 943)			
Step 1			
Depression	1.77 (1.37-2.27)	0.57	<.001
Contraceptive use	0.57 (0.42-0.78)	−0.55	.001
Depression × contraceptive use	NA	0.38	.09
Step 2			
Depression	1.02 (0.78-1.32)	0.02	.90
Contraceptive use	0.64 (0.46-0.89)	−0.44	.008
Depression × contraceptive use	NA	0.38	.10
Age	1.02 (1.01-1.03)	0.02	.001
Disease-related risk factors			
Smoking	1.37 (1.10-1.71)	0.31	.006
Hypertension	4.79 (3.76-6.11)	1.57	<.001
Hyperlipidemia	1.88 (1.48-2.39)	0.63	<.001
Diabetes	1.50 (1.16-1.95)	0.41	.002
DVT (n = 29 548)			
Step 1			
Depression	2.02 (1.48-2.75)	0.70	<.001
Contraceptive use	0.87 (0.60-1.24)	−0.14	.45
Depression × contraceptive use	NA	−0.39	.17
Step 2			
Depression	1.64 (1.20-2.25)	0.50	.002
Contraceptive use	0.98 (0.68-1.41)	−0.02	.90
Depression × contraceptive use		−0.35	.21
Age	1.02 (1.01-1.04)	0.02	.003
Disease-related risk factors			
Cancer	2.45 (1.87-3.21)	0.90	<.001
Obesity	1.52 (1.16-1.99)	0.42	.002
Thrombophilia	11.16 (6.60-18.86)	2.41	<.001
Antiphospholipid syndrome	1.15 (0.59-2.24)	0.14	.69

Abbreviations: DVT, deep vein thrombosis; MACE, major adverse cardiovascular events; NA, not applicable; OR, odds ratio.

**Table 3.** Associations of Anxiety and Contraceptive Use With MACE and DVT

Outcome, step, and variable	OR (95% CI)	*b*	*P* value
MACE (n = 27 142)			
Step 1			
Anxiety	1.59 (1.11-2.23)	0.47	.01
Contraceptive use	0.67 (0.53-0.85)	−0.40	.001
Anxiety × contraceptive use	NA	−0.06	.83
Step 2			
Anxiety	1.06 (0.74-1.51)	0.06	.76
Contraceptive use	0.75 (0.58-0.96)	−0.29	.02
Anxiety × contraceptive use	NA	−0.01	.96
Age	1.02 (1.01-1.03)	0.02	<.001
Disease-related risk factors			
Smoking	1.32 (1.06-1.64)	0.28	.01
Hypertension	5.22 (4.13-6.60)	1.65	<.001
Hyperlipidemia	1.80 (1.43-2.26)	0.59	<.001
Diabetes	1.52 (1.18-1.96)	0.42	.001
DVT (n = 28 694)			
Step 1			
Anxiety	2.07 (1.37-3.02)	0.73	<.001
Contraceptive use	0.79 (0.58-1.06)	−0.24	.12
Anxiety × contraceptive use	NA	−0.74	.04
Step 2			
Anxiety	1.74 (1.16-2.60)	0.55	.007
Contraceptive use	0.91 (0.67-1.23)	−0.10	.53
Anxiety × contraceptive use	NA	−0.69	.06
Age	1.03 (1.01-1.04)	0.03	<.001
Disease-related risk factors			
Cancer	2.39 (1.83-3.11)	0.87	<.001
Obesity	1.48 (1.15-1.92)	0.40	.003
Thrombophilia	11.31 (6.79-18.82)	2.43	<.001
Antiphospholipid syndrome	1.23 (0.64-2.36)	0.21	.53

Abbreviations: DVT, deep vein thrombosis; MACE, major adverse cardiovascular events; NA, not applicable; OR, odds ratio.

**Table 4.** Association of PTSD and Contraceptive Use With MACE and DVT

Outcome, step, and variable	OR (95% CI)	*b*	*P* value
MACE (n = 29 042)			
Step 1			
PTSD	1.36 (0.86-2.04)	0.31	.16
Contraceptive use	0.64 (0.51-0.80)	−0.45	<.001
PTSD × contraceptive use	NA	0.68	.03
Step 2			
PTSD	0.86 (0.55-1.34)	−0.15	.50
Contraceptive use	0.70 (0.55-0.88)	−0.36	.002
PTSD × contraceptive use	NA	0.69	.003
Age	1.02 (1.01-1.03)	0.02	<.001
Disease-related risk factors			
Smoking	1.28 (1.04-1.58)	0.25	.02
Hypertension	5.06 (4.04-6.35)	1.62	<.001
Hyperlipidemia	1.90 (1.52-2.37)	0.64	<.001
Diabetes	1.48 (1.16-1.88)	0.39	.001
DVT (n = 30 720)			
Step 1			
PTSD	1.45 (0.83-2.34)	0.37	.16
Contraceptive use	0.64 (0.48-0.84)	−0.44	.002
PTSD × contraceptive use	NA	0.26	.54
Step 2			
PTSD	1.50 (0.89-2.54)	0.41	.13
Contraceptive use	0.76 (0.57-1.01)	−0.28	.06
PTSD × contraceptive use	NA	0.19	.66
Age	1.03 (1.02-1.04)	0.03	<.001
Disease-related risk factors			
Cancer	2.32 (1.80-2.99)	0.84	<.001
Obesity	1.51 (1.18 to 1.94)	0.41	.001
Thrombophilia	10.49 (6.33 to 17.39)	2.35	<.001
Antiphospholipid syndrome	1.19 (0.63 to 2.27)	0.18	.59

Abbreviations: DVT, deep vein thrombosis; MACE, major adverse cardiovascular events; NA, not applicable; OR, odds ratio; PTSD, posttraumatic stress disorder.

**Table 5.** Model Estimates for Stratified Analyses Examining Hormonal Contraceptive Use and Major Adverse Cardiovascular Events Risk in Women With and Without PTSD, Adjusting for Clinical Covariates

Factor	PTSD (n = 1770)			No PTSD (n = 27 272)		
	OR (95% CI)	*b*	*P* value	OR (95% CI)	*b*	*P* value
Contraceptive use	1.68 (0.87-3.24)	0.52	.12	0.69 (0.54-0.87)	−0.38	.001
Age	1.05 (1.00-1.10)	0.05	.02	1.02 (1.01-1.03)	0.02	.003
Disease-related risk factors						
Smoking	1.26 (0.64-2.48)	0.23	.50	1.29 (1.03-1.62)	0.23	.04
Hypertension	9.41 (3.54-25.02)	2.24	<.001	4.84 (3.83-6.13)	1.58	<.001
Hyperlipidemia	1.94 (0.95-3.95)	0.66	.006	1.88 (1.48-2.38)	0.63	<.001
Diabetes	0.66 (0.32-1.37)	−0.42	.27	1.65 (1.28-2.13)	0.50	<.001

Abbreviations: OR, odds ratio; PTSD, posttraumatic stress disorder.

The only stress-related disorder that moderated associations between contraceptive use and MACE was PTSD (*b* = 0.69; *P* = .003). Analyses stratified by PTSD status revealed that hormonal contraceptive use was associated with approximately 31% lower odds of MACE among women without PTSD (Table 5); among women with PTSD, there was no association with hormonal contraceptive use, although the direction was as hypothesized (odds ratio, 1.68; 95% CI, 0.87-3.24; *P* = .12). Associations between contraceptive use and DVT risk as moderated by stress-related disorders were also mixed, with no significant associations when adjusting for multiple comparisons. Among women with single contraceptive generation prior to consent, there were no significant generation-specific moderating associations by depression, anxiety, or PTSD with MACE or DVT.

## Discussion

To our knowledge, this cohort study represents the largest investigation to date of associations between combined hormonal contraceptive use and cardiovascular and thrombotic outcomes in women with vs without stress-related disorders. Findings were mixed and largely contrary to hypotheses, in that there was insufficient evidence that the stress-related disorders of depression and anxiety moderated associations between contraceptive use and MACE and DVT risk. The 1 exception to this pattern of findings was PTSD; specifically, we observed lower MACE risk associated with contraceptive use in women without PTSD but not women with PTSD. These results may be considered initial evidence of the distinctiveness of PTSD. Finally, exploratory analyses considering contraception according to discrete generations revealed null findings irrespective of psychiatric disorders.

We observed differential findings for PTSD than for depression and anxiety. Specifically, we observed that PTSD status moderated the association between hormonal contraceptive use and MACE, with lower MACE risk in women without PTSD using contraceptives. In contrast, women with PTSD using contraceptives had greater estimated likelihood of MACE, but there was no statistically significant association. These preliminary findings could indicate that intermediate phenotypes of PTSD, such as impaired fear learning and increased sympathetic arousal, confer greater cardiovascular risk than features more associated with depression and anxiety (eg, behavioral inhibition or dysphoria). While depression and anxiety can also be characterized by sympathetic arousal, it is a more consistent feature of PTSD, where physiological reactivity to trauma reminders are primary manifestations of the disorder.^37,38^ Aligned with this, a prior study found that higher levels of re-experiencing symptoms, but not dysphoria, were associated with increased hypertension risk in women.^39^ Thus, hormonal contraceptive use may interact with these features to contribute to elevated cardiovascular risk (ie, MACE) in women with PTSD. It is also worth noting that differences in women with vs without PTSD may be attributable to unmeasured confounds, such as exposure to certain trauma types (eg, sexual assault) or lack of social and/or relational support.

Our findings, while preliminary, suggest a need for a greater clinical focus on PTSD and contraceptive use. Extant research has primarily studied these associations in the context of fear learning and extinction in experimental settings,^40,41^ findings that converge on the role of hormonal contraceptives in peritraumatic fear processing. These studies have demonstrated that women using hormonal contraceptives demonstrate enhanced fear acquisition and extinction^42^ and more optimal PTSD symptom profiles^43,44^—suggesting that there is something protective about hormonal contraceptive use in the context of PTSD. This interpretation is at odds with our findings, which imply that these protective associations may not extend to women’s cardiovascular health. This discrepancy requires additional examination.

Exploratory analyses disaggregating contraception into generations revealed null findings, with no evidence to support moderating associations of any of the stress-related disorders and associations with MACE or DVT. This contrasts with meta-analytic evidence supporting differential associations of contraceptive generations in relation to thrombotic risk^27^ and may be due to methodological differences. Because some women have histories of using multiple contraceptive generations (ie, transitioned to a third-generation contraceptive after using a second-generation method), we limited the exploratory analyses only to women with codes indicative of a single contraceptive generation prior to consent. While this categorization approach increases our confidence in the associations of discrete generations, it also greatly reduced our sample size. Future research in more controlled settings may be more well-equipped to tease apart differential associations.

### Limitations

While study strengths include data from an ecologically valid biobank, this work also has several limitations, many of which are inherent to EHR research.^45^ These include assumptions that any record in the EHR was indicative of a positive case for that study variable, and we relied on codes—which could have been entered in patient EHRs by any health care professional—to represent diagnoses vs gold standard clinical interviews; as such, bias due to diagnostic misclassification and variation in validity of diagnoses across practitioner types is possible. Nevertheless, this is an approach that has been adopted in other research on stress-related psychopathologies across biobanks.^31^ We were further constrained by what information is included in the EHR; for example, because disorder severity is inconsistently coded in medical records,^46^ we were unable to examine potential roles of psychiatric illness severity (eg, mild or severe) and course (eg, recurrent episode or chronic) across diagnoses. We also did not account for length of contraceptive use and amount of time between the stress-related diagnoses and the cardiovascular and thrombotic events. While these types of limitations are typical in EHR research, our findings should be interpreted in the context of the methodological constraints. Furthermore, as data were obtained from predominantly White, non-Hispanic patients seeking care at hospitals in the Boston area, our findings may not generalize to other racial or ethnic groups, nonclinical populations, and/or individuals in other geographic regions.

Moreover, base rates of both MACE and DVT were low (1.5% and 0.9% respectively), limiting statistical power necessary to detect potential associations. It is likely that these rates were influenced by features of the study design, such as excluding women older than 55 years, who are more likely to have experienced adverse cardiovascular and thrombotic events,^1,47^ and use of the consent date to account for temporality, which excluded women with MACE or DVT codes preceding stress-related diagnoses or contraception and truncated the number of years possible for a MACE or DVT event to occur. These methodological factors likely explain nonsignificant findings, as less than 0.1% of women coded positive on these 3 variables. MACE and DVT represent discrete diagnostic end points that are preceded by various clinical risk factors that can take years to lead to MACE and DVT (eg, hypertension, hypercoagulability, or endothelial dysfunction).^15,47^ As such, investigations that focus on hormonal contraception’s role in these intermediatory processes may be more informative and thus represent an important avenue for future research. Additional mechanistic work could also consider examining disorders (including those events constituting MACE here) as discrete outcomes, in service of better understanding potentially differential pathways leading from contraception to various CVD events.

## Conclusions

In this cohort study of 31 824 women enrolled in large, hospital-based biobank, we found preliminary evidence that hormonal contraceptive use contributed to the impact of specific stress-related psychopathologies—namely, PTSD—on adverse cardiovascular outcomes. Despite methodological limitations of EHR research, findings highlight the importance of considering contraceptive use in the context of women’s mental and cardiac health, particularly in women with PTSD. Future research disentangling the role of hormonal contraceptive use in women with PTSD and potential outcomes on cardiovascular risk mechanisms is warranted.

## Notes

Supplement 1. eTable. RxNorm codes for combined hormonal contraceptives eFigure. Selection criteria used for study cohort

Supplement 2. Data Sharing Statement
