# Supplementary material for: Hormonal Contraceptive Use, Stress Disorders, and Cardiovascular and Thrombotic Risk in Women
Source: JAMA Netw Open. 2026 Jan 2;9(1):e2551878. doi: 10.1001/jamanetworkopen.2025.51878 (PMC12761333; doi:10.1001/jamanetworkopen.2025.51878)
Supplement: Supplement 1. — eTable. RxNorm codes for combined hormonal contraceptives eFigure. Selection criteria used for study cohort [file jamanetwopen-e2551878-s001.pdf]

Supplemental Online Content

Thomas JL, Ellis RA, Karam K, et al. Hormonal contraceptive use, stress disorders, and cardiovascular and thrombotic risk in women. *JAMA Netw Open.* 2026;9(1):e2551878. doi:10.1001/jamanetworkopen.2025.51878

eTable. RXNORM codes for combined hormonal contraceptives

eFigure. Selection criteria used for study cohort

This supplemental material has been provided by the authors to give readers additional information about their work.

© 2026 Thomas JL et al. *JAMA Network Open*.

eTable. RXNORM codes for combined hormonal contraceptives

Category	Description	RXNORM
**Hormonal Contraceptives**		
First-generation	Estrogens/medroxyprogesterone	1006917
	Ethinyl estradiol/ethynodiol	214557
	Ethinyl estradiol/norethindrone	384410
	Mestranol/norethindrone	154643
Second-generation	Ethinyl estradiol/levonorgestrel	214558
	Ethinyl estradiol/norgestrel	37075
Third-generation	Desogestrel/ethinyl estradiol	324044
	Ethinyl estradiol/etonogestrel	352376
	Ethinyl estradiol/norelgestromin	352377
	Ethinyl estradiol/norgestimate	214559
Fourth-generation	Dienogest/estradiol	994203
	Drospirenone/ethinyl estradiol	840781
	Drospirenone/ethinyl estradiol/levomefolate	1013625

© 2026 Thomas JL et al. *JAMA Network Open*.

eFigure. Selection criteria used for study cohort

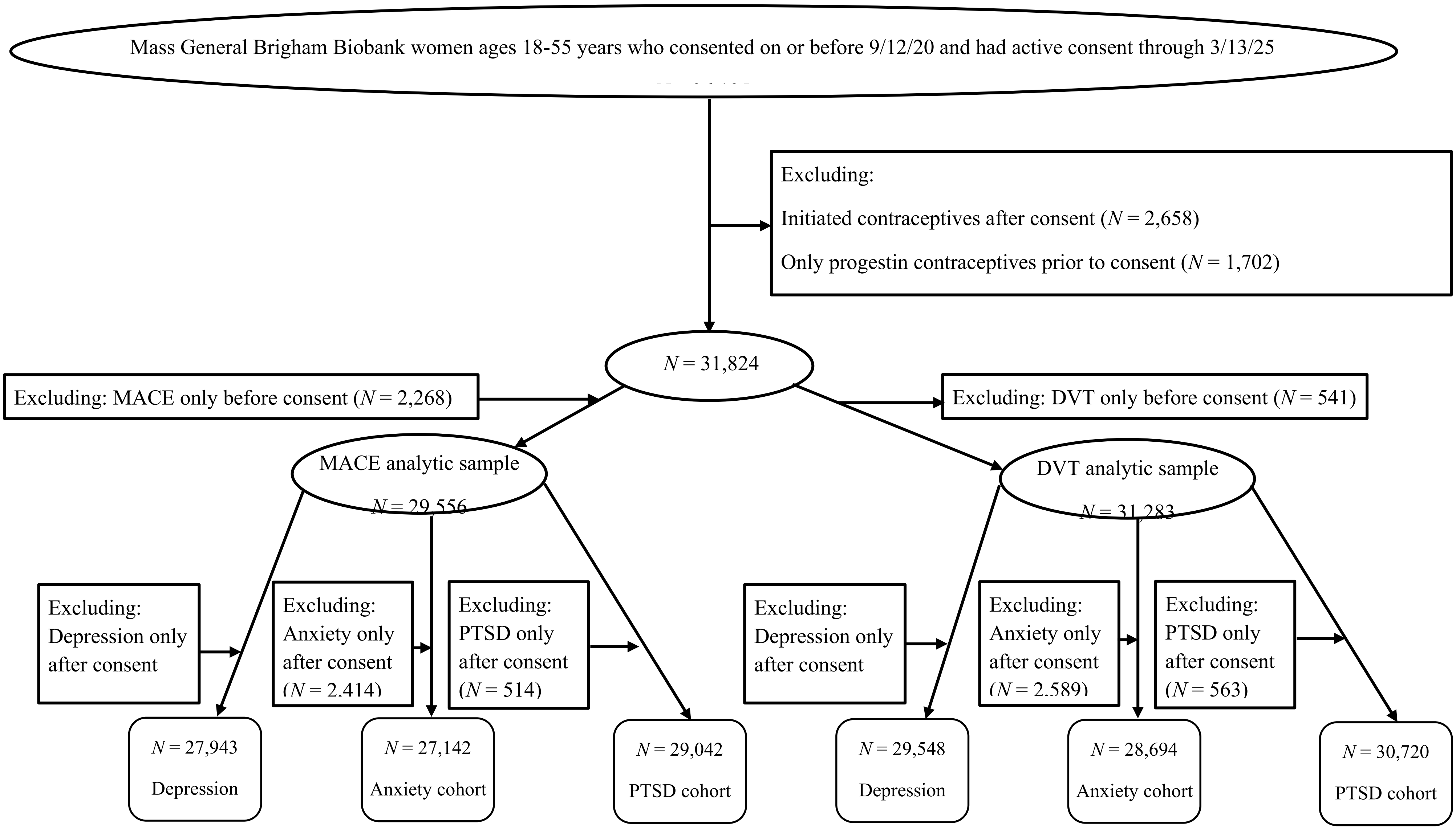

© 2026 Thomas JL et al. *JAMA Network Open*.
