# Supplementary material for: Hormonal Contraceptive Use, Stress Disorders, and Cardiovascular and Thrombotic Risk in Women
Source: JAMA Netw Open. 2026 Jan 2;9(1):e2551878. doi: 10.1001/jamanetworkopen.2025.51878 (PMC12761333; doi:10.1001/jamanetworkopen.2025.51878)
Supplement: Supplement 2. — Data Sharing Statement [file jamanetwopen-e2551878-s002.pdf]

Data Sharing Statement

Thomas. Hormonal Contraceptive Use, Stress Disorders, and Cardiovascular and Thrombotic Risk in Women. *JAMA Netw Open*. Published January 02, 2026.
doi:10.1001/jamanetworkopen.2025.51878

Data
Data available: No

Additional Information
Explanation for why data not available: Biobank patient data
